# Mushroom-Derived Novel Selenium Nanocomposites’ Effects on Potato Plant Growth and Tuber Germination

**DOI:** 10.3390/molecules27144438

**Published:** 2022-07-11

**Authors:** Olga M. Tsivileva, Alla I. Perfileva

**Affiliations:** 1 Laboratory of Microbiology, Institute of Biochemistry and Physiology of Plants and Microorganisms, Saratov Scientific Centre of the Russian Academy of Sciences, 13 Prospekt Entuziastov, 410049 Saratov, Russia; 2 Laboratory of Plant-Microbe Interactions, Siberian Institute of Plant Physiology and Biochemistry, Siberian Branch of the Russian Academy of Sciences, 664033 Irkutsk, Russia; alla.light@mail.ru

**Keywords:** *Solanum tuberosum* L., higher fungi, basidiomycetes, selenium, biocomposites, growth stimulating properties, *Clavibacter sepedonicus*, *Cms*, antiphytopathogenic activity

## Abstract

Multicomponent materials, where nanosized selenium (Se) is dispersed in polymer matrices, present as polymer nanocomposites (NCs), namely, selenium polymer nanocomposites (SeNCs). Selenium as an inorganic nanofiller in NCs has been extensively studied for its biological activity. More ecologically safe and beneficial approaches to obtain Se-based products are the current challenge. Biopolymers have attained great attention with perspective multifunctional and high-performance NCs exhibiting low environmental impact with unique properties, being abundantly available, renewable, and eco-friendly. Composites based on polysaccharides, including beta-glucans from edible and medicinal mushrooms, are bioactive, biocompatible, biodegradable, and have exhibited innovative potential. We synthesized SeNCs on the basis of the extracellular polysaccharides of several medicinal mushrooms. The influence of bio-composites from mushrooms on potato plant growth and tuber germination were studied in two potato cultivars: Lukyanovsky and Lugovskoi. Bio-composites based on *Grifola umbellata* demonstrated the strongest positive effect on the number of leaves and plant height in both cultivars, without negative effect on biomass of the vegetative part. Treatment of the potato tubers with SeNC from *Gr. umbellata* also significantly increased germ length. Potato plants exposed to Se-bio-composite from *Ganoderma lucidum* SIE1303 experienced an increase in the potato vegetative biomass by up to 55% versus the control. We found earlier that this bio-composite was the most efficient against biofilm formation by the potato ring rot causative agent *Clavibacter sepedonicus (Cms)*. Bio-composites based on *Pleurotus ostreatus* promoted increase in the potato root biomass in the Lugovskoi cultivar by up to 79% versus the control. The phytostimulating ability of mushroom-based Se-containing bio-composites, together with their anti-phytopathogenic activity, testifies in favor of the bifunctional mode of action of these Se-biopreparations. The application of stimulatory green SeNCs for growth enhancement could be used to increase crop yield. Thus, by combining myco-nanotechnology with the intrinsic biological activity of selenium, an unexpectedly efficient tool for possible applications of SeNCs could be identified.

## 1. Introduction

The development of novel nanostructured materials using renewable natural resources is a promising approach within the green biotechnology area. Among the contemporary challenges in biological synthesis of nanomaterials are, unquestionably, investigations into the vast potentialities of medicinal and nutritional mushrooms in bioproduction of functional nanosized conjugates for such fields as healthcare, food packaging and processing, environment, and agriculture [1,2,3]. Owing to a broad set of applications of nanoparticles (NPs), fungal-assisted fabrication of nanomaterials has attracted the attention of scientists to a novel area of research, myco-nanotechnology [4], a new term that was proposed originally by Mahendra Rai et al. [5]. Commercially versatile, highly metal tolerant, easy to handle, with large biomass and metabolite yield, mushroom cultures are well suited for the production of a wide range of nanoparticle types [6], including selenium NPs (SeNPs). Selenium is an essential nutrient that regulates a variety of cellular processes in every living organism [7,8]. This microelement in humans encourages the immune system to perform its critical protective function and demonstrates powerful anticancer and antimicrobial effects [9]. By combining myco-nanotechnology with the intrinsic biological activity of selenium, unexpectedly efficient tools for feasible applications of SeNPs could be identified, because of their appealing physicochemical and functional properties [10,11]. Moreover, the use of nanomaterials allows high doses of selenium to be avoided, while retaining biological effects [12].

More ecologically safe and beneficial approaches to obtaining Se-comprising products are of urgent necessity. SeNPs exhibit significant antibacterial activity in both non-capped [13,14] and conjugated forms, such as the SeNPs-lysozyme nanohybrid system [15]. Conjugates with edible mushroom biopolymers, yielding selenium nanocomposites (SeNCs), should be environmentally friendly and offer an economically beneficial mode for using microelement selenium compounds in plant growth and protection. Features common to natural polysaccharides are low toxicity, high biodegradability, biocompatibility, and bio-adhesivity [16,17]. For instance, the anti-inflammatory activity of natural polysaccharide-modified SeNPs has been proved [18,19]. Mushrooms’ polysaccharides, mainly extracellular glucans, are capable of serving as capping and stabilizing agents for NPs, hence resulting in better dispersed, homogeneous and uniformly-sized SeNPs. These biopolymeric shells for SeNPs prevent clustering of the latter and provide them with useful properties [20]. At the beginning of our studies, we looked at the available literature, and we did not find any information on the production of SeNCs using mushroom cultures.

The most promising applications of myco-synthesized nanomaterials in agriculture include, among others, the development of nano-formulations for crop yield improvement and for control of phytopathogenic infections [21]. Bulky doses of agrochemicals are required by conventional agricultural processes, adversely affecting ecosystems. Application of myco-nanotechnology in sustainable agricultural practices could diminish the large-scale production of plant-growth promoters, fertilizers, pesticides, etc., by enhancing the efficiency of agricultural inputs. Therewith, the involvement of safe myco-chemicals, based on optimized consistent products that can be easily extracted and separated in sufficient quantities from mushroom cultures, is extremely important. To gain harvest abundance and quality in principle strategic crops, including potatoes (*Solanum tuberosum* L.), grown extensively throughout the world [22], the aforesaid environmentally friendly preparations are desirable to incorporate both phytostimulating and pesticidal properties. In particular, the potato suffers severely from a phytopathogenic Gram-positive bacterium *Clavibacter michiganensis* ssp. *sepedonicus* (*Cms*) (Spiekermann and Kotthoff, 1914; Davis et al., 1984), designated presently as *Clavibacter* *sepedonicus* [23], called the potato ring rot causative agent [24]. Efficient suppressing strategies for this bacterium are actually lacking at present [25,26]. Moreover, attempts to restrict the expansion of potato bacterial diseases via ecologically acceptable chemical or biological means have failed, and the application of biocontrol agents remains highly problematic [27,28]. Tremendous ecological risks are also associated with the increasing resistance of phytopathogens to chemical pesticides [29].

Hence, an actual need exists for developing nano-formulations based on natural compounds efficient in combating pathogenic bacteria, eco-friendly to plants, and possibly beneficial for potato health and stimulatory to its growth. Biopolymeric composites of fungal origin could be the most satisfactory solution. Our focus of interest in this regard was on hybrid organic–inorganic bio-composites containing potentially growth promoting and antimicrobial components, the bioproduction of which are mediated by extracellular fungal metabolites, as we reported earlier [30]. Antibacterial activity of the resulting SeNCs against *Cms* was revealed [31], and the dependence of the antimicrobial effect on the mushroom’s taxonomic position was explored [32]. The current work aimed at elucidating the effect of nutritional and medicinal mushrooms on potato growth characteristics of the ecologically safe biopreparations we developed earlier. The developed SeNCs were explored in comparative studies with analogously synthesized Se-free bio-compositions, and their impact on plant morphological parameters, as well as on potato tuber germination, was evaluated.

## 2. Results and Discussion

### 2.1. SeNCs’ Features

The produced mushroom-assisted SeNCs were selenium-containing polyglucans with a mass portion of Se 0.002–0.005%, depending on the fungal species [33,34]. The identity of the SeNPs was verified by the results of dynamic light scattering (DLS) analysis, in accordance with which the average particle size of SeNPs was found to be 254 nm, and electrophoretic light scattering (ELS), according to which the values of zeta potential were determined to be about −40.8 mV (Figure S1). The biosynthesized SeNPs’ surface charge was depicted by a zeta potential of a high absolute value (Supplementary Materials, Figure S1B). Many research groups have established that the surface charge of (nano)structures serves as a critical parameter important to their stability [35]. Current studies reported in literature that have dealt with the biogenic synthesis of different negatively charged NPs testify to their high stability and biological activity [36]. The presence of biomolecules adjacent to, and surrounding, NPs of different chemical natures present a principal reason for changes in their zeta potential quantity [37].

The biosynthesized SeNPs’ size distribution in a sample solution (Figure S1A) was determined by means of the DLS method and depicted the hydrodynamic diameter of the particles. The DLS method estimates the measured NPs as bigger compared to the same objects evaluated by electron microscopy techniques [38]. Application of the Scanning Electron Microscopy (SEM) technique, coupled with Energy-Dispersive X-ray Spectroscopy (EDXS), enabled visualization of the SeNPs in the form of white objects, which were close-to-spherically shaped, and were fairly evenly distributed in the polymeric biomatrix (Figure S2). Indeed, the SeNPs’ average size was lesser (150 nm) than that detected within the framework of dynamic light scattering (254 nm), where the SeNPs’ diameter was influenced by the stabilizing capping biomolecules on their surface.

Powder X-ray diffraction (XRD) pattern analysis demonstrated a broad pattern analogous to [6]. XRD did not confirm crystallinity of the myco-synthesized SeNPs, or, to be more precise, of the selenium phase in the compositions under question because of this method’s inability to distinguish between different the oxidation states of selenium. This behavior of relevant reflections lacking in X-ray diffractograms testified to the fact that SeNPs were assembled from the amorphous “red” allotropic modification of this chemical element (Figure S3). A dark-red color of Se-phase was also in line with the presence of a much more biologically active red amorphous Se(0), compared to inert grey crystalline selenium [39]. The same was confirmed by the studies using a Raman spectroscopy method [40]. The spectroscopic Raman characteristics of mycogenically formed and harvested Se(0) spheres were the signal intensity values (in arbitrary units) at different Raman shift quantities (in cm^−1^) in Raman spectra [41]. Our results compared favorably with previously described findings for abiotically formed Se(0) of different allotropic modifications, and with findings gained from analyses of biogenic production of amorphous Se(0) by bacteria [42,43] or blue-green microalga [44]. Low-molecular-weight substances, as presumable admixtures in the composition of SeNCs, could, in principle, be detectable within the framework of high-performance liquid chromatography (HPLC) [45], but were not revealed. That picture was also true for another chromatographic method used, namely, the gas chromatography-mass spectrometry (GC-MS) technique. The results regarding the yield of the typical reaction medium, after fungal culturing with Se-supplementation (Figure S4B), were very similar to the GC-MS yield profile of the Se-free reaction medium, after fungal culturing without the Se-supplementation (Figure S4A). One could conclude there was an absence of any appreciable GC-MS-detected low-molecular-weight substances as admixtures to SeNCs’ chemical composition.

Mushroom metabolite-assisted biotransformation of organic Se-precursor compound into Se(0) phase was followed by occurrence of the SeNPs accompanied by the wrapping of NPs with a fungal extracellular polymeric biomatrix. Thus, the role of the biopolymeric envelope consisted in providing a sustained dispersion of SeNPs and giving rise to formation of nanostructures without their apparent clustering, enabling the consequent formation of bio-composites. The fact that the major extracellular polysaccharidic molecules should be responsible for reduction and subsequent stabilization during the formation of SeNPs, was observed with a number of polyglucans and inorganic Se-precursor compounds in the course of physicochemical processes; for example, γ-irradiation of solutions containing Se^4+^ ions and water-soluble yeast β-glucan, resulted in synthesis of SeNPs [46]. Fourier transform infrared (FTIR) spectroscopy revealed that β-glucan could interact with SeNPs through linkages between selenium and the oxygen atoms that are abundant in polysaccharide, resulting in a homogeneous and semitransparent solution. Chemically and phytochemically synthesized SeNPs, capped with polyglucans, were recently revealed to show an immunostimulant activity [46,47], and to display strong antibacterial and antifungal effects [48,49].

### 2.2. Biocompositions Effect on Potato Plant Growth

**Figure 1:**
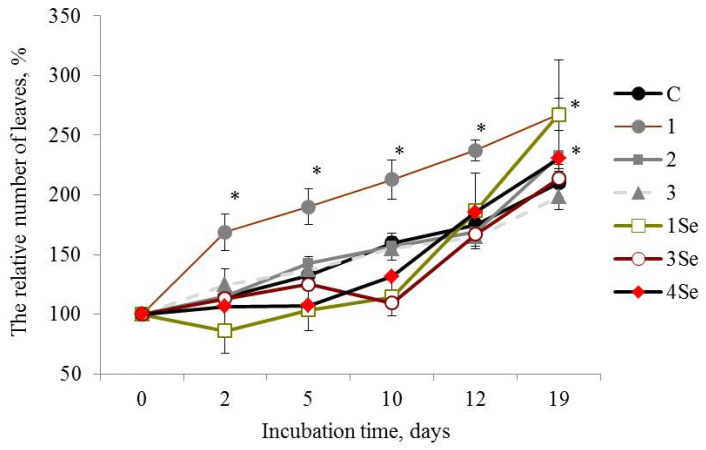
Influence of bio-composites on the relative (percentage to “0 day”) number of plant leaves in Lukyanovsky potato cultivar in vitro. C—control without treatment by fungal bio-composites; 1, 2 and 3—treatment by fungal biopreparations without Se based on *Grifola umbellata*, *Ganoderma lucidum* 1315 and *Pleurotus ostreatus*, respectively; 1Se, 3Se and 4Se—treatment by fungal bio-composites with Se based on *Gr. umbellata*; *P. ostreatus* and *G. lucidum* SIE1303; * *p* ≤ 0.01 compared to control.

Plants of the Lukyanovsky potato cultivar exposed to the Se-free bio-composition, based on *Gr. umbellata*, performed the best in studies of this cultivar, judging from such parameters as number of leaves (Figure 1) and plant height (Table 1). Seven-day-old plants treated with this preparation possessed a number of leaves exceeding those of the untreated control plants by more than 1.5 times, the latter value remaining not less than 1.25 times greater compared to the control over the entire observation period (Figure 1).

Plant height indices for the Lukyanovsky potato cultivar exposed to the Se-free preparation from *Gr. umbellata* exceeded the corresponding values of control plants within the whole duration of the experiment, with the exception of the 3rd and 25th days of incubation, the maximal increase being 17% (Table 1).

Selenium-containing fungal bio-composites exerted positive impact on the number of plant leaves in the Lukyanovsky potato cultivar, particularly after twelve days of growth on the SeNC-supplemented media (Figure 1). Over the rest of the observation period, the treatment by SeNCs, based on *Gr. umbellata*, *P. ostreatus* and *G. lucidum* SIE1303, caused increase in the parameter of number of plant leaves, that exceeded the untreated control maximally by 1.3 times in the case of Se-biopreparation based on *Gr. umbellata* (Figure 1(1Se)).

**Figure 2:**
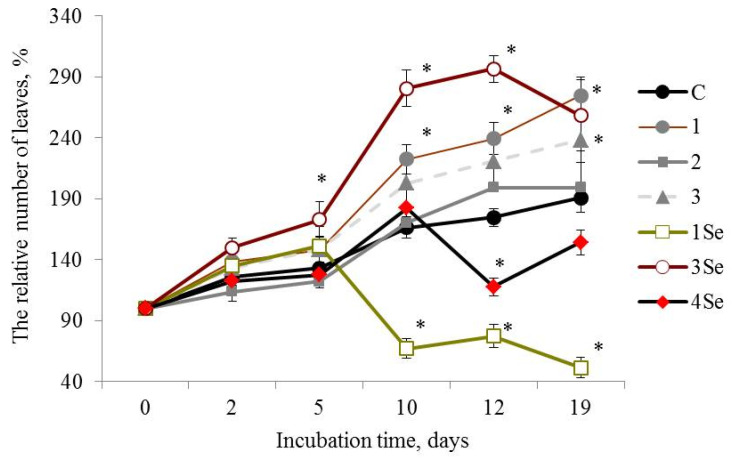
Influence of bio-composites on the relative (percentage to “0 day”) number of plants leaves in Lugovskoi potato cultivar in vitro. C—control without treatment by fungal bio-composites; 1, 2 and 3—treatment by fungal biopreparations without Se based on *Grifola umbellata*, *Ganoderma lucidum* 1315 and *Pleurotus ostreatus*, respectively; 1Se, 3Se and 4Se—treatment by fungal bio-composites with Se based on *Gr. umbellata*; *P. ostreatus* and *G. lucidum* SIE1303; * *p* ≤ 0.01 compared to control.

As for the Lugovskoi potato cultivar exposed to the Se-free fungal preparations, the plants treated with the composition from *Gr. umbellata* performed the best in the whole study, judging by the number of leaves (Figure 2(1)) and plant height morphometric quantities (Table 1). However, research into the influence of other bio-composites, not presented in Table 1, on height of in vitro plants, exhibited a rather poor yield from potato treatment by fungal biopreparations without Se based on *G. lucidum* SIE1303, as well as by fungal bio-composites with Se based on *Gr. umbellata*; *P. ostreatus* and *G. lucidum* SIE1303 (Figure S5).

At the 19th day after the experiment start, plants of the Lugovskoi potato cultivar treated with the Se-free *Gr. umbellata* preparation showed that the parameter of the number of leaves number was more than 1.4 times greater than the control plants. Plant height values in this given mode of experiment (Table 1) constantly increased with culture duration, and were more than two times greater in comparison with control from the 5th day of growth and over the entire observation period, the maximal ratio being equal to 2.64 for 21-day-old plants. Consequently, a favorable impact from *Gr. umbellata* biopreparations, in which fungal polysaccharides were an essential ingredient, obviously took place in the above assays. The edible-and-medicinal mushroom *Grifola umbellata* (Pers.) Pilát, also termed *Polyporus umbellatus* (Pers.) Fries, belongs to Polyporaceae, Basidiomycetes, and is distributed widely over most areas of East Asia, Europe and North America [50]. The therapeutic properties of *Gr. umbellata* polysaccharides was demonstrated in the course of clinical studies [51,52]. Bi et al. [53] firstly reported not only antitumor and immunomodulatory effects, but also antioxidant properties of two polysaccharides isolated from *Gr. umbellata*. All mushroom polysaccharides are known for their own specific compositions and structural details [54]. Glucose was stated to be the principal constituent monosaccharide, with small amounts of other sugar residues, like mannose, fucose, xylose, galactose [55,56]. The varying monosaccharide compositions complemented the minor constituents, such as uronic acid and protein in *Gr. umbellata* metabolites [51,53], and led to unique product biosynthesis in *Gr. umbellata*, revealing an intrinsic stimulatory capacity in potato plants.

Plant height values for the Lugovskoi potato cultivar exposed to the *P. ostreatus*-based Se-free bio-composition exhibited a relatively moderate increase in growth (Table 1). Nevertheless, by the end of this period, the plants were about 1.5 times longer versus the control potato. Furthermore, even though the Se-free biopreparation obtained from the mushroom *P. ostreatus* was not a majorly effective stimulatory agent in respect of both cultivars under study, it was highly superior in accelerating the growth of potato plants from the Lugovskoi cultivar, compared to the Lukyanovsky one (Table 1).

*G. lucidum* 1315-based Se-free bio-composition (Figure 2(2)) and SeNCs obtained from *Gr. umbellata*, *G. lucidum* SIE1303 (Figure 2(1Se,4Se)) revealed mainly weak positive or even negative effects on the parameter of number of leaves of the Lugovskoi potato cultivar plants. The above weak positive action was only either before 5 days or after 10 days of culture duration. When considering the influence of other bio-composites on the relative (percentage to untreated control) number of plant leaves in both potato cultivars in vitro, one could note a rather poor yield from the potato treatment by fungal biopreparations without Se based on *G. lucidum* SIE1303, as well as by fungal bio-composites with Se based on *Ganoderma lucidum* 1315 (Figure S6). In contrast, the impact of *P. ostreatus*-based Se-bio-composite on the aforesaid morphometric index in plants from the Lugovskoi potato cultivar was highly positive (Figure 2(3Se)), and in 2 weeks after the experiment began, the value of the number of leaves exceeded control by more than 1.6 times. However, the effect essentially decreased by the end of the observation period.

Therefore, the in vitro vegetation experiment revealed that the Lukyanovsky potato cultivar plants susceptible to the ring rot causative agent were also rather susceptible to the mushroom-originating Se-containing bio-compositions. The latter’s positive action in respect to the parameter of the number of plant leaves was observed within a growth period from 12 days to the end of the experiment, accompanied by the most profound effect of the SeNC based on *Gr. umbellata*, which was 1.3 times superior, relative to control. The Lugovskoi potato cultivar plants selected for their resistance to the biotrophic pathogen *Cms*, were less susceptible to the Se-containing bio-composites in respect to the parameter of the number of plant leaves. Only when using the *P. ostreatus*-based SeNC, was a remarkably positive action exerted; however, it tended to decrease by the end of the observation period.

The important traits in estimating the effect of different phytostimulants are commonly recognized to include shoot and root mass [57]. We evaluated the quantities of plant material biomass, which appeared to be distinct for different mushroom producents in the bio-compositions used for potato treatment. The mass of the vegetative parts (Table 2) of the Lukyanovsky potato cultivar plants increased as a result of exposure to Se-biopreparation based on *G. lucidum* SIE1303 (plus 32% to control), and Se-free bio-compositions obtained from *P. ostreatus*, *Gr. umbellata* (plus 10% and 4% to control, respectively). However, the effect of the fourth Se-free preparation based on *G*. *lucidum* SIE1303 (Figure S7(4-a,4-b)) along with SeNCs based on *Grifola umbellata* (Figure S7(1Se-a,1Se-b)) and *Ganoderma lucidum* 1315 (Figure S7(2Se-a,2Se-b)) remained well below the corresponding control values (Figure S7(C-a,C-b)).

Remarkable enlargement of the values of plant vegetative part biomass in the Lugovskoi potato cultivar was again observed with the *G*. *lucidum* SIE1303-SeNC (plus 55% to control) along with the fungal agents without Se, i.e., bio-compositions based on *G. lucidum* 1315 and *Gr. umbellata* (plus 18% and 27% to control, respectively). However, at all the just mentioned experiment modes implementing Lugovskoi potato cultivar, the plant root biomass quantities were less than those in untreated plants (0.86–0.91 to control).

Meanwhile, promising results were exhibited by the *P. ostreatus*-derived biopreparations, both SeNC and Se-free. The root biomass values in the Lugovskoi potato cultivar plants exposed to these bio-compositions increased by 1.64 and 1.79 times compared to control, respectively. The vegetative part biomass quantities reached an undoubtedly acceptable level, exceeding control by 1.18 and 1.40 times, respectively (Table 2). A prerequisite for better yield and quality of potatoes, as an extremely popular crop worldwide, is closely related to the physiological processes of the plant parts, both underground and aboveground. The yield of tubers as the most valuable part of this crop is not solely dependent on the physiological and morphological indices of the aboveground parts of the plants. It was important that, for the plants undergoing the *P. ostreatus*-derived biopreparations, not only the vegetative part mass, but also the plant root mass increased. Oyster mushroom (*P. ostreatus*) is known by its pronounced antioxidant properties [58,59]. Therefore, it is likely that the effect of oyster mushroom metabolites implemented in production of the fungal bioagent would exert a stimulatory effect on potato plants with subsequent enhancement of biomass accumulation.

### 2.3. Biocompositions’ Effects on Potato Tubers Germination

**Figure 3:**
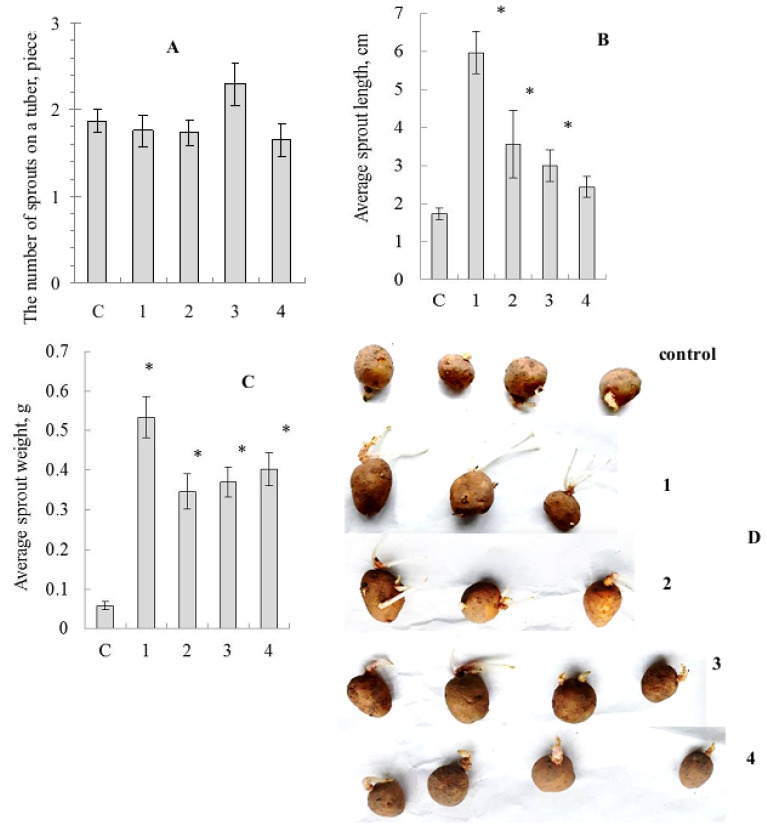
Influence of treatment by the mushroom-based Se-nanocomposites on potato tuber germination. (**A**)—mean number of sprouts per tuber, (**B**)—mean sprout length, (**C**)—mean sprout biomass, (**D**)—photo of tubers; c—control (distilled water), 1—*Grifola umbellata*, 2—*Ganoderma lucidum* 1315, 3—*Pleurotus ostreatus*, 4—*G. lucidum* SIE1303; * *p* ≤ 0.01 compared to control.

The effect of seed tuber pre-treatment by SeNCs on potato germination was revealed. The results of such treatment were clearly understood, even in visual observation of the tubers (Figure 3).

Biometric parameter assessment showed no significant differences in average number of sprouts for tubers treated by SeNCs, compared to the distilled water-treated control, although there was some superiority of the oyster mushroom-derived SeNC (Figure 3A). Nevertheless, the average sprout length values were stimulated under the action of SeNCs, and the maximal effect was caused by the potato tuber pre-treatment with SeNC based on *Gr. umbellata* (Figure 3B). The average sprout biomass quantities were remarkably enhanced (Figure 3C), as influenced by all the mushroom-produced SeNCs under the study, compared to control.

### 2.4. Biocompositions’ Prospects in Relation to Antibacterial Properties

In this work, the most efficient Se-nanocomposite to combat biofilm formation by the potato ring rot causative agent, was that based on *Ganoderma lucidum* SIE1303, which facilitated increase in potato plant vegetative part mass (Table 2). One of the antimicrobial effects of chemical or biological agents developed to combat plant pathogens is their ability to counteract the formation of biofilms by the pathogenic bacteria [60]. Biofilms developed by *Cms* within the plant vascular system are regarded as the main cause of wilting of potato leaves [61].

For more efficient and ecologically safe applications of the mushroom-originated preparations described in this study, the results obtained should be correlated to the antimicrobial effect of the SeNCs we revealed earlier, depending on the biological species of mushroom, and which culture was used for the bio-composite synthesis [30,62]. The impact of SeNCs based on *G. lucidum*, *Gr. umbellata*, *P. ostreatus*, and some other macromycetes, on the viability and biofilm-forming capacity in *Cms* was studied. Bacterium viability impairment in the fungal Se-preparations was shown to be caused by the *Cms* cells’ incubation. A decisive role for decrease in viability was ascribed to just the Se(0) component of SeNCs. Mushroom taxonomic position contributed moderately to the antibacterial effect of Se-biopreparations. The most pronounced activity among the macromycetes studied in the present work was revealed for the bio-composites based on the extracellular metabolites of *G. lucidum* 1315, and for the strain *G. lucidum* SIE1303, which exhibited the capability of inhibiting bacterial biofilm formation; which was also found in [32]. The biochemical action of the mushroom *G. lucidum* made a significant contribution to the anti-biofilm-forming efficiency of bio-composites. The interest in developing various nutraceuticals and functional products from *Ganoderma* genus permanently grows [63]. This has encouraged researchers to study the species *Ganoderma* from various geographical origins to facilitate the identification of natural compounds with pronounced antibacterial, antioxidant, medical and other useful properties. A distinctive feature of the metabolites pool produced by *Ganoderma* are triterpenes, including ganoderic acids [64], which have antimicrobial properties and which are capable of adversely affecting the *Cms* biofilm occurrence. These low molecular weight compounds formed by *G. lucidum* are involved in the constructing steps of the bio-compositions, and could be regarded as prerequisites of the antibiofilm-forming properties of the biopreparations based on this mushroom.

## 3. Materials and Methods

### 3.1. SeNCs’ Physicochemical Studies

Biopolymeric compositions were synthesized on the basis of the extracellular metabolites of the mushroom cultures *Ganoderma lucidum* 1315, *Ganoderma lucidum* SIE1303, *Grifola umbellata* 1622, and *Pleurotus ostreatus* HK352, grown by means of the submerged culture technique on synthetic media for 28 days at 27 °C in the presence of an organic Se-containing substrate diacetophenonyl selenide (1,5-diphenylselenopentanedione-1,5, bis(benzoylmethyl)selenide, preparation DAPC-25, Sulfat, Russia [65]), as well as on the same media without Se-additives. Mushroom metabolites transformed this Se-precursor substance into elemental Se (Se(0)) [39] for making further bio-composites [66].

The size distribution assessment and zeta potential measurement of the prepared SeNPs were carried out using a Zetasizer Nano ZS instrument (Malvern Instruments Ltd., Worcestershire, UK). The SeNCs’ morphologies were visualized by a Scanning Electron Microscopy (SEM) method using a Mira II LMU scanning electron microscope (Tescan, Brno, Czech Republic). Prior to measurements, the samples were sputter-coated with carbon and then metalized with gold to provide each sample with the best conductive properties [67]. The SEM images constructed by detecting electrons back-scattered from the sample, not secondary electrons, were considered optimal for SeNCs. Chemical element distribution in SeNCs was analyzed with an Energy-Dispersive X-ray Spectroscopy (EDXS) method using a EDX INCA Energy 350 system (Oxford Instruments, Witney, UK) by mapping the signal of an element [68,69]. Powder X-ray diffraction (XRD) pattern analysis using the DRON-3 diffractometer (Burevestnik, Moscow, Russia), strengthened by the Raman spectroscopy analysis completed on a Ntegra Spectra system (NT-MDT, Zelenograd, Russia), were carried out to reveal whether the Se(0) phase was crystalline or amorphous, and to elucidate an allotropic modification of selenium [39,40,70]. Selenium mass portion in the SeNCs obtained was determined by the atomic absorption spectroscopy method [71]. Gas chromatographic analysis of the minor chemical component levels in the bio-compositions was conducted in the mass-spectrometric detection mode (GC-MS) using a Trace GC-DSQ mass spectrometer system (Thermo Finnigan, San Jose, CA, USA) [72]. Chromatographic analysis was also performed with a Shimadzu HPLC system (Shimadzu Corporation, Kyoto, Japan) in the high-performance liquid chromatography (HPLC) mode to detect the biomolecules of mushroom cultures involved in the fabrication of SeNPs [62].

### 3.2. Cultivation of Plants In Vitro

In vitro *Solanum tuberosum* L. plants of Lugovskoi and Lukyanovsky potato cultivars (All-Russian Research Institute of Potato Breeding n.a. A.G. Lorkh), which were resistant and susceptible to *Cms*, respectively [73,74], were used. Micro-clonal reproduction of plants was carried out using cuttings. The cuttings were placed at the internode depth into an agar nutrient Murashige and Skoog (MS) medium (4.2 g/L, pH 5.8–6.0), supplemented with vitamins and hormones, and cultivated for 14 days at a temperature of 24–25 °C, illumination of 5–6 kLux, photoperiod of 16 h; and then transferred into a liquid MS medium of the same composition, but without agar. This liquid growth medium of potato was supplemented with an aqueous solution of the bio-composition under study at a concentration equivalent to a mass portion of Se 0.000625% in the culture medium. This was attained in order to be consistent with A.I.P. previous research dealing with non-fungal polymeric matrices for SeNCs [75]. Afterwards, the plants were grown on these liquid bio-composition-containing media for 28 days with a periodic evaluation of shoot growth increment. Biometric indicator assessment, such as number of leaves and plant height, was repeated every 2–3 days, and mass quantities of vegetative part (VP) and roots were recorded at the end of each experiment, as commonly accepted [76]. Fresh biomass of the potato plant material under question was measured gravimetrically [77].

### 3.3. Tuber Sprouting Experiments

In the course of studies of the effects of biopreparations on potato tuber germination, tubers were treated with the bio-compositions’ aqueous solutions by spraying, and stored in a dark chamber at +18 °C for 14 days. Then, the following parameters were counted and evaluated: number of sprouts on each tuber, the average length of sprouts from the tuber, and average sprout mass, according to recommendations [78,79].

### 3.4. Statistical Processing of Data

Statistical data processing was carried out using the SigmaPlot v.12.5 program (SYSTAT Software, Chicago, IL, USA). The data obtained after treatment were statistically compared with controls using the nonparametric Mann–Whitney *U* test. All values were expressed as the arithmetic mean with standard deviation (M ± SD), a *p*-value of less than 0.05 was considered significant.

## 4. Conclusions

This study contributes to a set of prerequisites regarding the introduction of selenium-containing bio-composites from the metabolites of mushrooms as a new, valuable and environmentally safe treatment in agriculture. The revealed phytostimulating properties of the bio-compositions based on *Gr. umbellata*, *G. lucidum* and *P. ostreatus* in respect to potato plants and tuber germination, amplified by substantial sensitivity to the potato ring rot causative agent, indicated that these fungal, bifunctional preparations, which are effective at very low doses, are natural, eco-friendly stimulants of potato plant growth, and tuber germination, and provide the means for possible improvement in potato plant health against bacterial disease. Further studies, including studies dealing with the utilization of mushrooms for the sustainable production of SeNCs, are needed to determine whether myco-synthesized SeNPs can be used as a promising tool for biological control and as substitutes for toxic synthetic chemical pesticides. We highlighted in detail the results, which are most useful for practice, and are capable of naturally reflecting the detailed features of the studied biological objects. Not all the objects, only two or three of the four mushrooms taken into consideration, appeared to fit our expectations in the whole set of experiments. However, one should recognize that in summary all four mushrooms used in SeNC synthesis satisfied our expectations in distinct terms, either in effect on plant height, number of plant leaves, vegetative part mass, root mass, combating bacterial phytopathogen biofilm formation, or in action on potato tuber germination. The work offers the possibility of reconciling the valorization of an abundant biomass with the development of agri-food products, while respecting the environmental nanotechnological framework.

## Figures and Tables

**Table 1:** Height of in vitro plants of Lukyanovsky and Lugovskoi potato cultivars in control (cm) and after treatment by fungal biopreparations without Se based on *Grifola umbellata* (1) and *Pleurotus ostreatus* (3) (percent of “0 day” in italic, and percent to control in bold).

Treatment	Incubation Time, Day												
	0	3	5	7	10	13	15	17	19	21	23	25	28
Lukyanovsky													
Control	5.43 ± 1.35	5.90 ± 1.18	6.43 ± 1.19	6.77 ± 1.45	7.63 ± 1.44	8.53 ± 2.11	9.10 ± 2.11	10.13 ± 1.96	10.40 ± 1.83	11.33 ± 2.40	12.20 ± 2.51	12.63 ± 2.85	17.20 ± 3.39
	*100*	*109.49 ± 7.07*	*119.71 ± 9.46*	*125.50 ± 12.86*	*142.04 ± 12.52*	*157.01 ± 5.17*	*167.92 ± 4.51*	*188.26 ± 10.76*	*193.78 ± 15.19*	*209.88 ± 7.87*	*226.30 ± 11.26*	*233.43 ± 5.54*	*320.21 ± 29.45*
1	*100*	*104.16 ± 4.59*	*137.46 ± 5.13*	*145.73 ± 12.79*	*166.54 ± 22.63*	*178.08 ± 20.59*	*195.68 ± 25.68*	*208.64 ± 27.40*	*213.55 ± 32.34*	*224.79 ± 30.77*	*229.40 ± 32.30*	*231.75 ± 30.66*	*324.28 ± 32.21*
	**100**	**95**	**115**	**116**	**117**	**113**	**117**	**111**	**110**	**107**	**101**	**99**	**101**
3	*100*	*102.07 ± 2.98* *	*90.65 ± 6.05*	*99.61 ± 7.84*	*120.88 ± 8.63*	*134.38 ± 11.02*	*147.92 ± 16.79*	*161.03 ± 21.17*	*164.16 ± 25.32*	*172.88 ± 22.39*	*177.71 ± 19.86*	*179.66 ± 20.24*	*231.79 ± 44.16*
	**100**	**93**	**76**	**79**	**85**	**86**	**88**	**86**	**85**	**82**	**79**	**77**	**72**
Lugovskoi													
Control	5.27 ± 0.23	5.57 ± 0.12	5.70 ± 0.35	6.13 ± 0.67	6.87 ± 0.60	8.00 ± 0.66	9.03 ± 0.71	9.80 ± 1.00	10.33 ± 1.55	11.00 ± 1.00	11.23 ± 1.05	11.60 ± 0.78	14.93 ± 1.05
	*100*	*105.90 ± 7.01*	*108.57 ± 11.63*	*116.99 ± 18.22*	*130.86 ± 17.21*	*152.44 ± 19.23*	*172.12 ± 21.19*	*186.81 ± 26.92*	*197.23 ± 37.94*	*209.63 ± 27.88*	*214.10 ± 29.14*	*220.79 ± 21.96*	*283.90 ± 22.96*
1	*100*	*128.94 ± 37.94*	*238.12 ± 18.32*	*260.70 ± 20.33*	*318.19 ± 26.15*	*388.04 ± 32.68*	*449.55 ± 37.60*	*491.07 ± 41.83*	*503.37 ± 43.09*	*553.70 ± 46.60*	*554.52 ± 45.72*	*556.72 ± 44.72*	*691.02 ± 55.99*
	**100**	**122**	**219**	**223**	**243**	**255**	**261**	**263**	**255**	**264**	**259**	**252**	**243**
3	*100*	*121.23 ± 19.37*	*124.10 ± 23.07*	*146.63 ± 20.71*	*172.91 ± 23.26*	*209.49 ± 28.96*	*226.60 ± 20.96*	*261.11 ± 34.54*	*273.33 ± 37.37*	*298.37 ± 39.22*	*314.31 ± 30.53*	*324.12 ± 36.37*	*432.65 ± 46.77*
	**100**	**114**	**114**	**125**	**132**	**137**	**132**	**140**	**139**	**142**	**147**	**147**	**152**

* *p* ≤ 0.01 compared to control.

**Table 2:** Biomass (mg) of roots and vegetative part (VP) of in vitro plants of Lukyanovsky and Lugovskoi potato cultivars in control and after 28 days of treatment by fungal biopreparations without Se based on *Grifola umbellata* (1), *Ganoderma lucidum* 1315 (2), *Pleurotus ostreatus* (3), and by fungal bio-composites with Se based on *P. ostreatus* (3Se), *G. lucidum* SIE1303 (4Se) (percent to control in parentheses).

Treatment	Lukyanovsky		Lugovskoi	
	Roots	VP	Roots	VP
Control	532 ± 71	559 ± 193	425 ± 62	482 ± 90
1	313 ± 88	584 ± 178	366 ± 86	612 ± 31
	(59)	(104)	(86)	(127)
2	498 ± 110	334 ± 102	387 ± 91	569 ± 121
	(94)	(60)	(91)	(118)
3	326 ± 122	614 ± 127	760 ± 142 *	677 ± 127
	(61)	(110)	(179)	(140)
3Se	374 ± 54	474 ± 129	697 ± 59	569 ± 103
	(70)	(85)	(164)	(118)
4Se	292 ± 39 *	739 ± 69	376 ± 81	747 ± 117 *
	(55)	(132)	(88)	(155)

* *p* ≤ 0.01 compared to control.

## Data Availability

Not applicable.

## Supplementary Materials

The following supporting information can be downloaded at: https://www.mdpi.com/article/10.3390/molecules27144438/s1, Figure S1: **A**—representative profile of SeNPs size distribution obtained by dynamic light scattering, **B**—zeta potential of the synthesized SeNPs; Figure S2: SEM image of the mycosynthesized Se-nanocomposite (Mira \\ LMU, Saratov State University); Figure S3: X-ray diffraction results confirming rather amorphous nature of the mycosynthesized SeNPs. Abscissa is for 2Theta values, and ordinate is for counts; Figure S4: Chromatogram (GC-MS) of the mycosynthesized SeNCs. **A**—profile of the Se-free reaction medium yielded after fungal culturing without Se-supplementation, **B**—profile of the reaction medium yielded after fungal culturing with Se-supplementation; Figure S5: Influence of biocomposites on the relative (percentage to “0 day”) height of in vitro plants in Lugovskoy potato cultivar. **C**—control without treatment by fungal biocomposites; 4—treatment by fungal biopreparation without Se based on *G. lucidum* SIE1303; 1Se, 3Se and 4Se—treatment by fungal biocomposites with Se based on *Gr. umbellata*; *P. ostreatus* and *G. lucidum* SIE1303, respectively; * *p* ≤ 0.01 compared to control, ** *p* ≤ 0.05 compared to control; Figure S6: Influence of biocomposites on the relative (percentage to”0 day”) number of plant leaves in Lukyanovsky (**a**) and Lugovskoy (**b**) potato cultivars in vitro. **C**—control without treatment by fungal biocomposites; 4—treatment by fungal biopreparation without Se based on *G. lucidum* SIE1303; 2Se—treatment by fungal biocomposite with Se based on *Ganoderma lucidum* 1315; *p* ≤ 0.05 compared to control; Figure S7: Influence of biocomposites on the relative (percentage to untreated control) biomass of roots and vegetative part (VP) of plants of Lukyanovsky (**a**) and Lugovskoy (**b**) potato cultivars in vitro after 28 days of treatment by fungal biopreparations without Se based on *G. lucidum* SIE1303 (4), and by fungal biocomposites with Se based on *Gr. umbellata* (1Se), *Ganoderma lucidum* 1315 (2Se).

## Sample Availability

Samples of the plant material and SeNCs are available from the authors.
