# Supplementary material for: Mushroom-Derived Novel Selenium Nanocomposites’ Effects on Potato Plant Growth and Tuber Germination
Source: Molecules. 2022 Jul 11;27(14):4438. doi: 10.3390/molecules27144438 (PMC9321743; doi:10.3390/molecules27144438)
Supplement: Supplementary file 1 [file molecules-27-04438-s001.zip › molecules-1756360-supplementary.pdf]

Supplementary Materials

Mushroom-Derived Novel Selenium Nanocomposites' Effects on Potato Plant Growth and Tuber Germination

Olga M. Tsivileva [1,*,†] and Alla I. Perfileva [2,†]

[1] Laboratory of Microbiology, Institute of Biochemistry and Physiology of Plants and Microorganisms, Saratov Scientific Centre of the Russian Academy of Sciences, 13 Prospekt Entuziastov, 410049 Saratov, Russia
[2] Laboratory of Plant-Microbe Interactions, Siberian Institute of Plant Physiology and Biochemistry, Siberian Branch of the Russian Academy of Sciences, 664033 Irkutsk, Russia; alla.light@mail.ru
* Correspondence: tsivileva_o@ibppm.ru
† These authors contributed equally to this work.

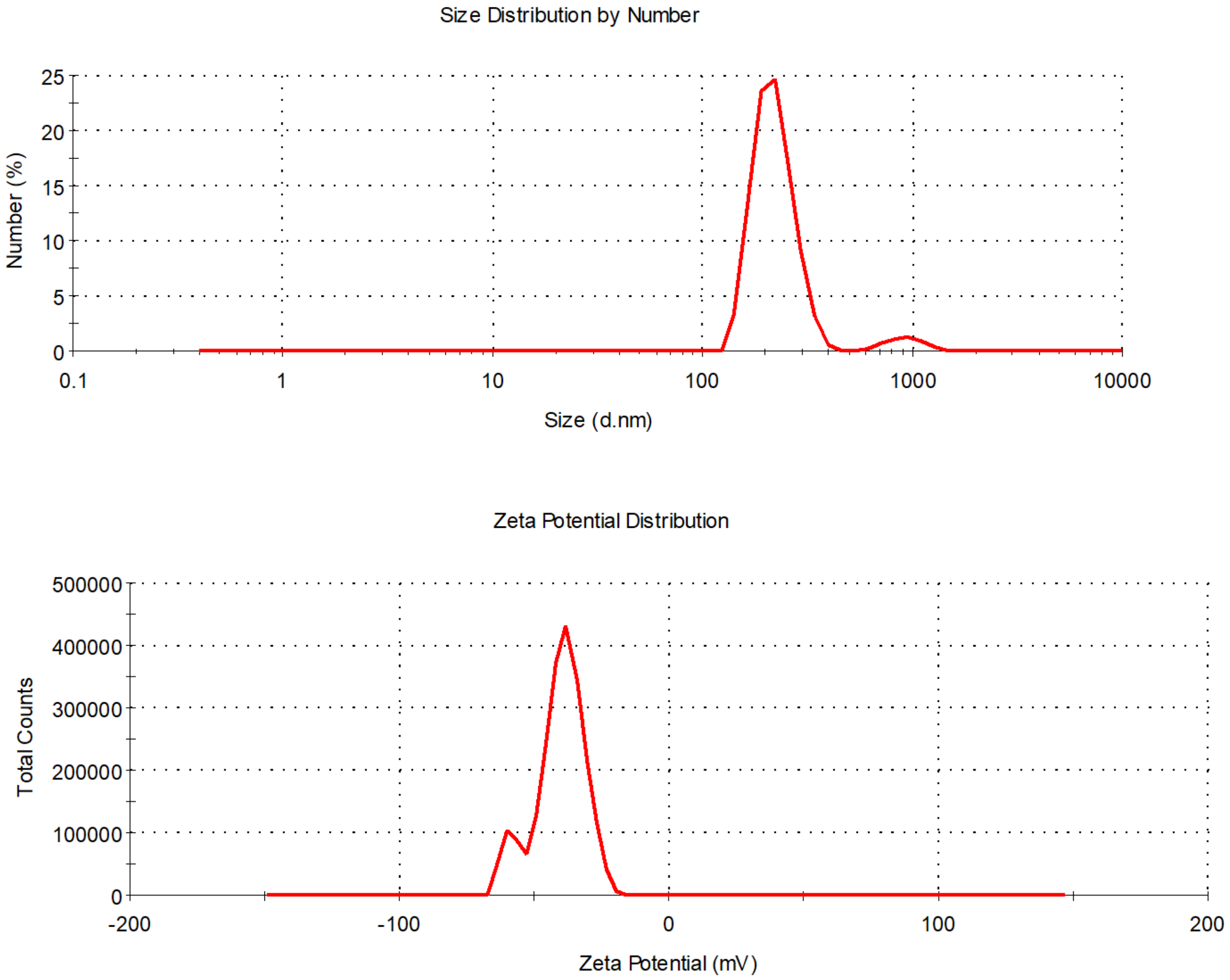

A

B

Figure S1. A—representative profile of SeNPs size distribution obtained by dynamic light scattering, **B**—zeta potential of the synthesized SeNPs.

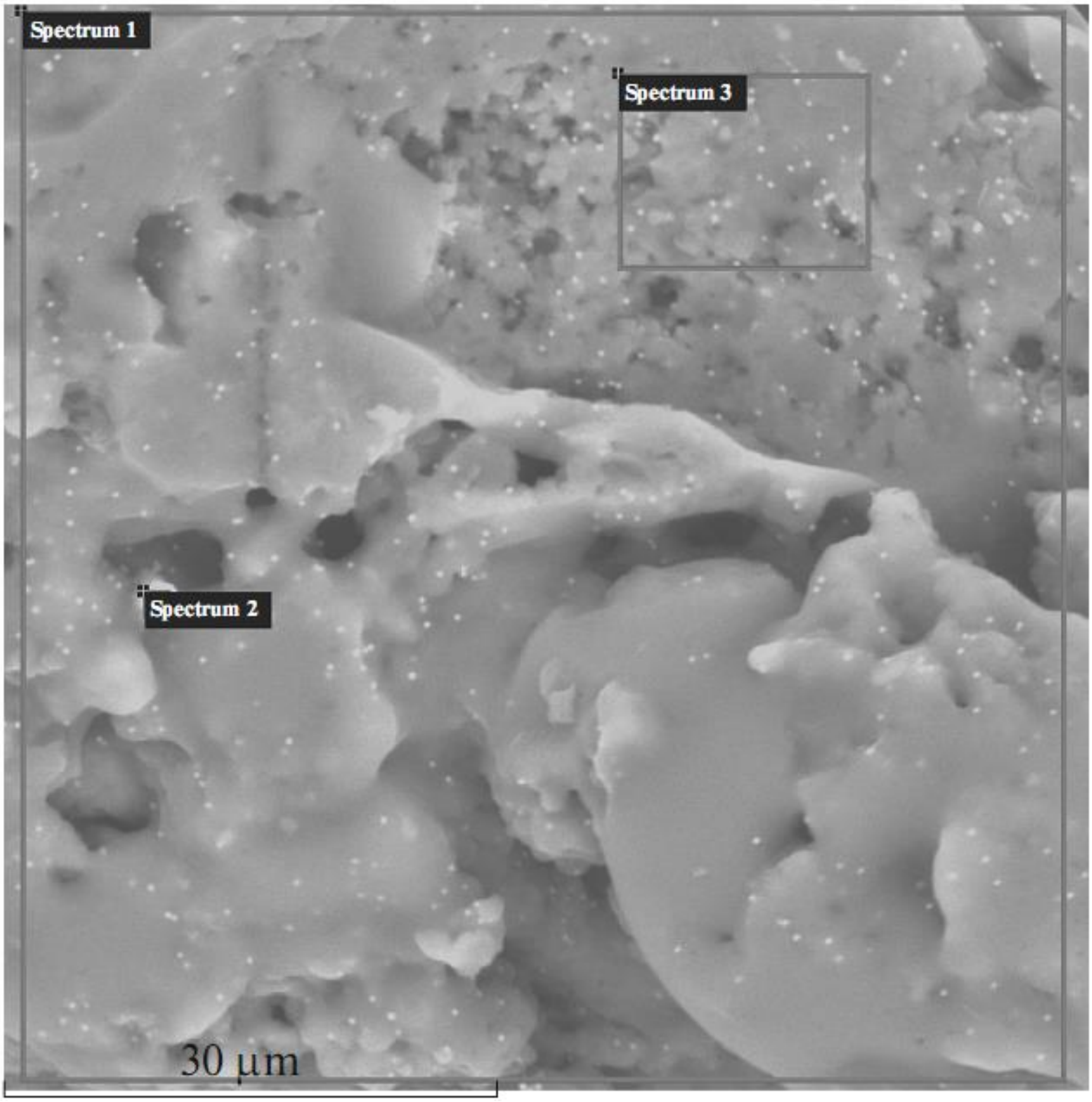

Figure S2. SEM image of the mycosynthesized Se-nanocomposite (Mira \\ LMU, Saratov State University).

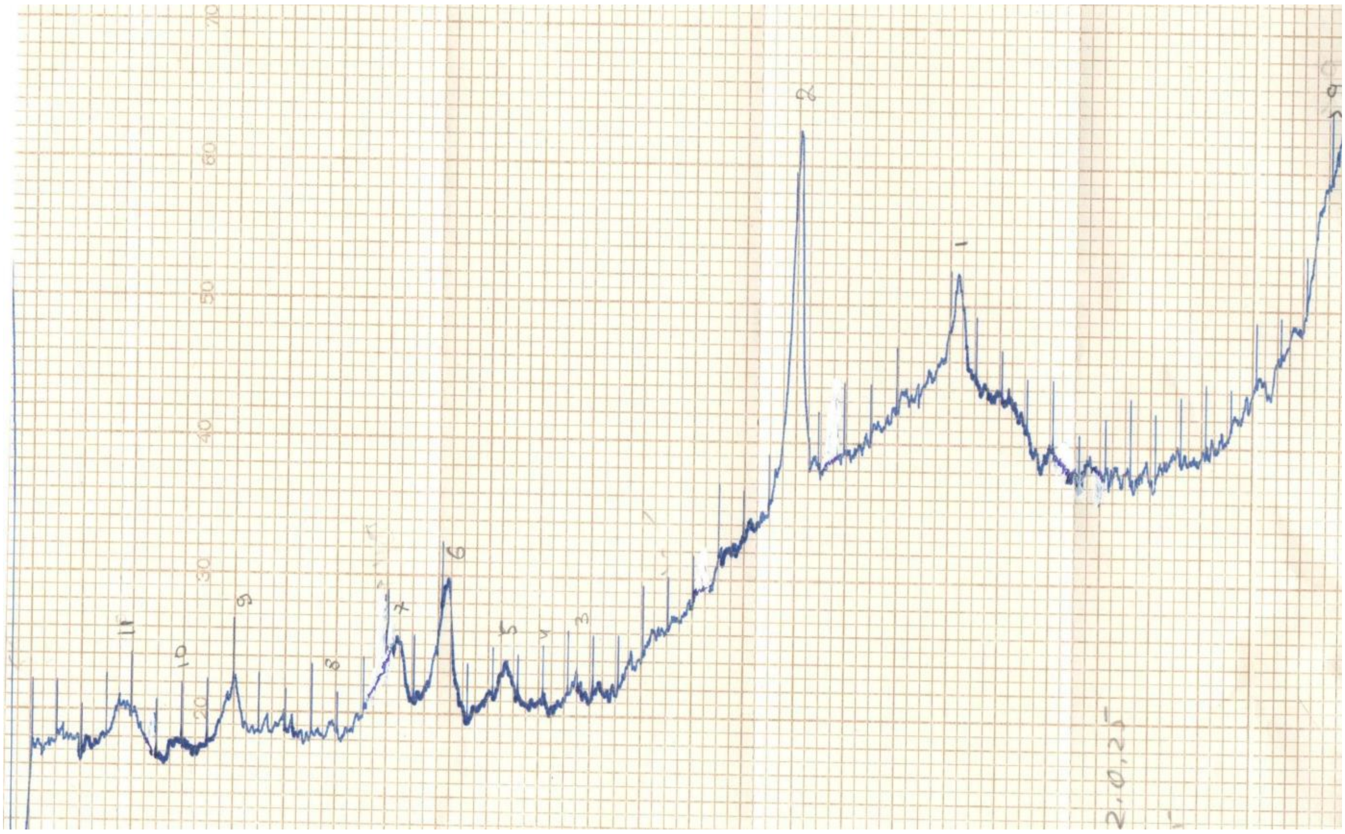

Figure S3. X-ray diffraction results confirming rather amorphous nature of the mycosynthesized SeNPs. Abscissa is for 2Theta values, and ordinate is for counts.

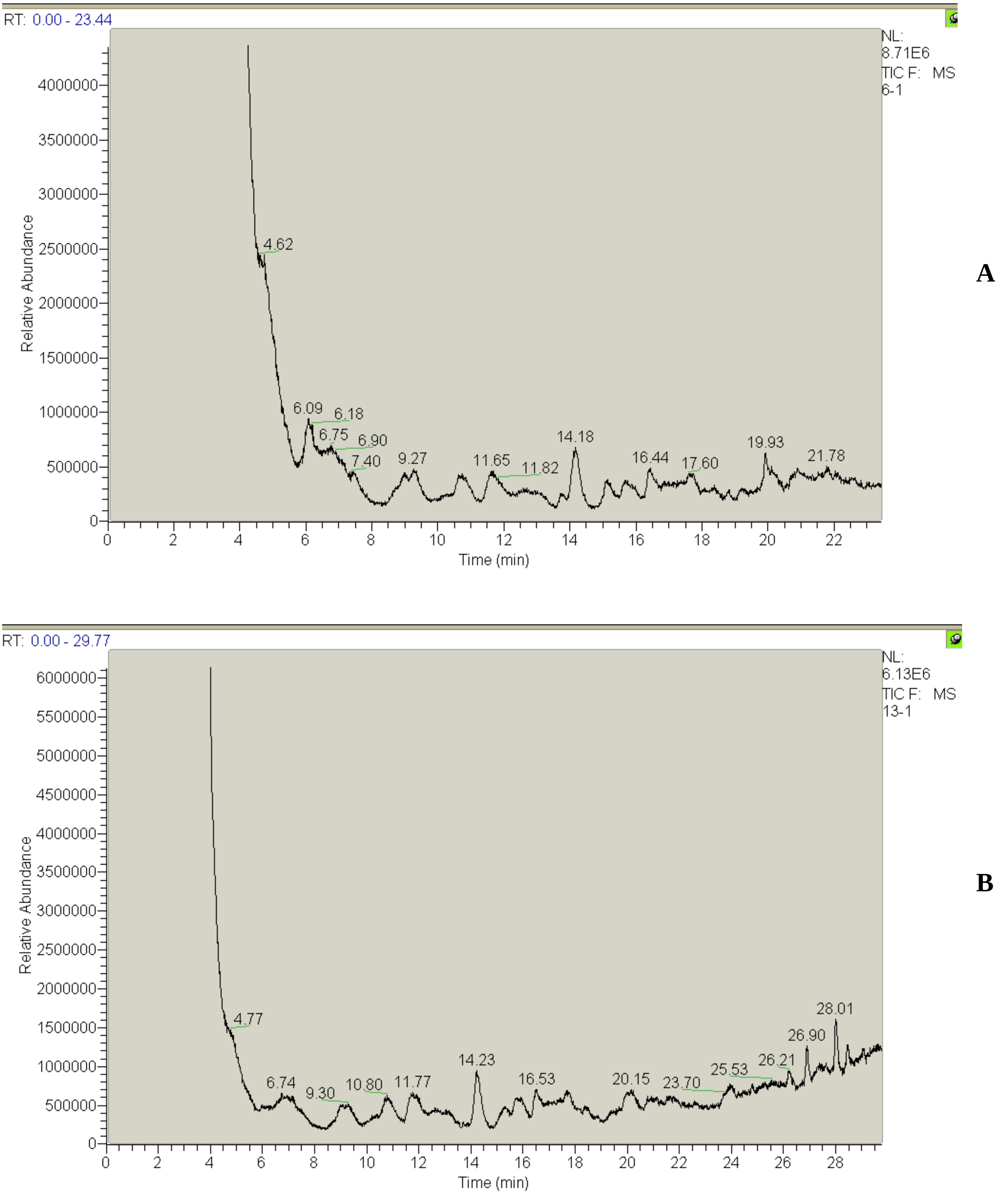

Figure S4. Chromatogram (GC-MS) of the mycosynthesized SeNCs. **A**—profile of the Se-free reaction medium yielded after fungal culturing without Se-supplementation, **B**—profile of the reaction medium yielded after fungal culturing with Se-supplementation.

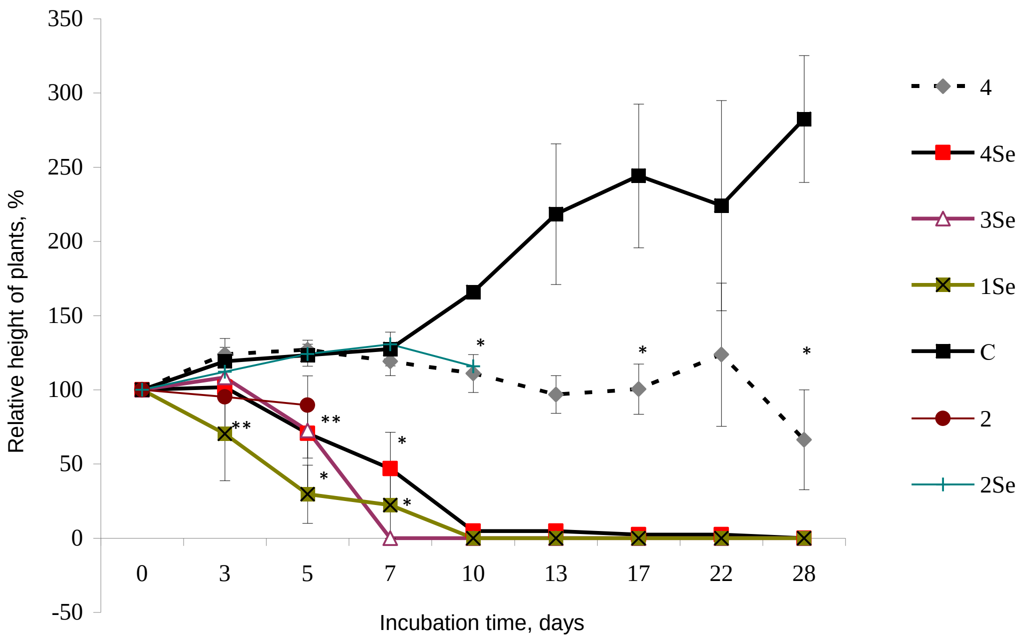

Figure S5. Influence of biocomposites on the relative (percentage to "0 day") height of in vitro plants in Lugovskoy potato cultivar. C—control without treatment by fungal biocomposites; 4—treatment by fungal biopreparation without Se based on *G. lucidum* SIE1303; 1Se, 3Se and 4Se - treatment by fungal biocomposites with Se based on *Gr. umbellata*; *P. ostreatus* and *G. lucidum* SIE1303, respectively; * $p \leq 0.01$ compared to control, ** $p \leq 0.05$ compared to control

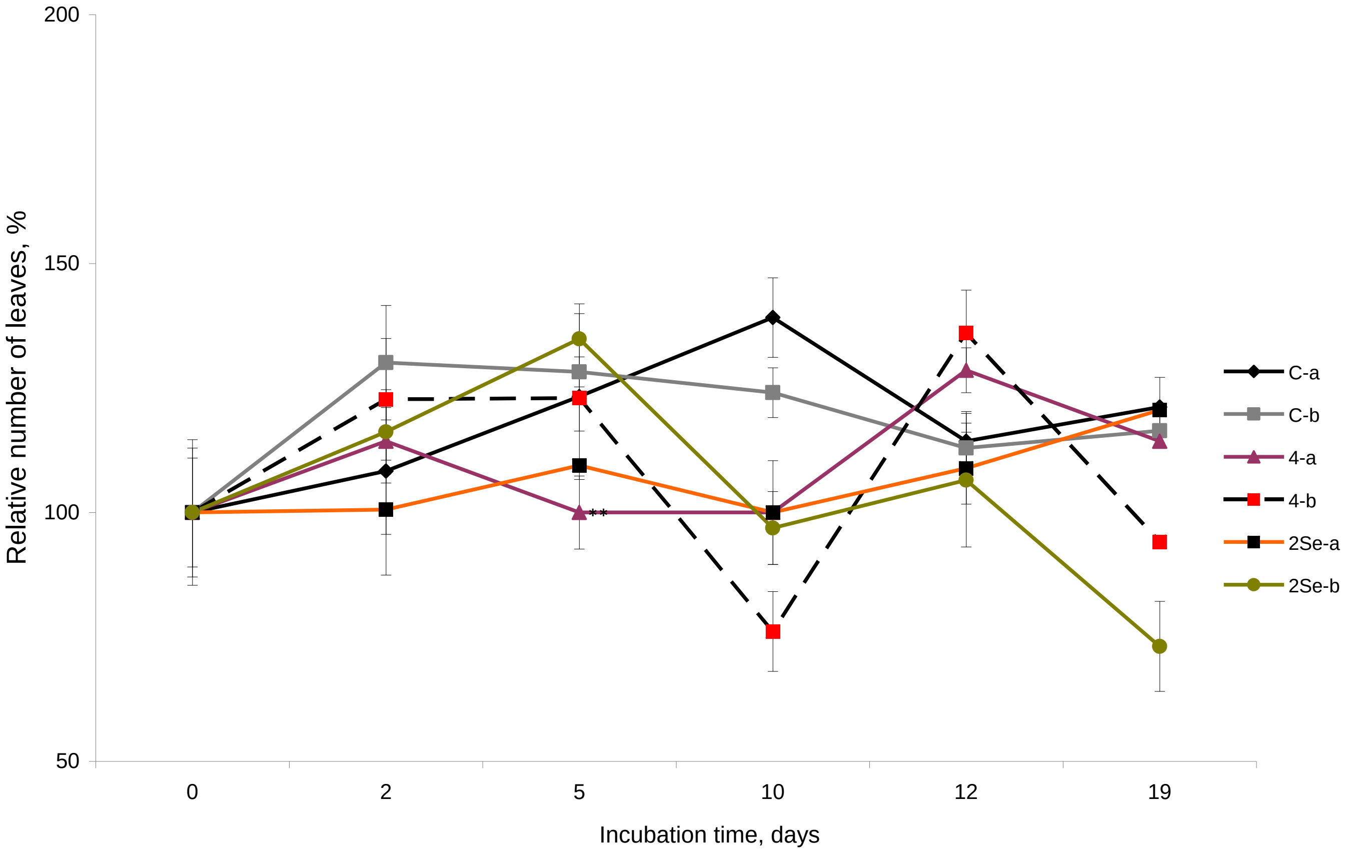

Figure S6. Influence of biocomposites on the relative (percentage to"0 day") number of plant leaves in Lukyanovsky (**a**) and Lugovskoy (**b**) potato cultivars in vitro. C—control without treatment by fungal biocomposites; 4—treatment by fungal biopreparation without Se based on *G. lucidum* SIE1303; 2Se—treatment by fungal biocomposite with Se based on *Ganoderma lucidum* 1315; $p \leq 0.05$ compared to control

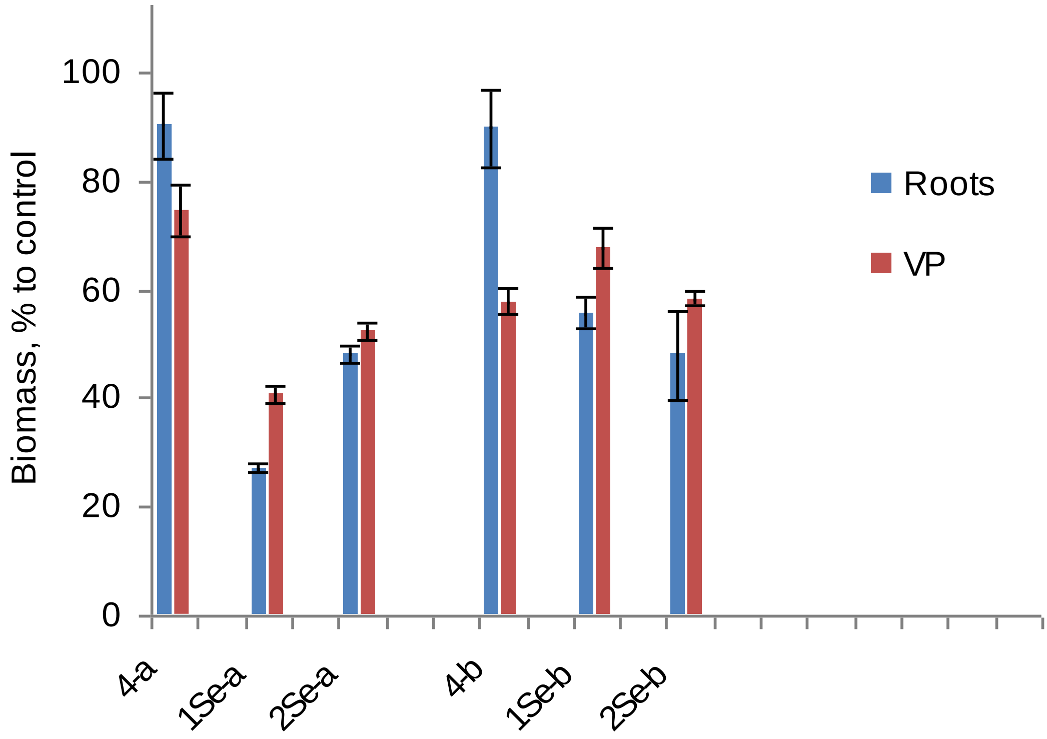

Figure S7. Influence of biocomposites on the relative (percentage to untreated control) biomass of roots and vegetative part (VP) of plants of Lukyanovsky (**a**) and Lugovskoy (**b**) potato cultivars in vitro after 28 days of treatment by fungal biopreparations without Se based on *G. lucidum* SIE1303 (4), and by fungal biocomposites with Se based on *Gr. umbellata* (1Se), *Ganoderma lucidum* 1315 (2Se).
